# Isolation of *Toxoplasma gondii* from the placenta of northern fur seals (*Callorhinus ursinus*) and potential transplacental transmission of the parasite

**DOI:** 10.1051/parasite/2025045

**Published:** 2025-08-01

**Authors:** Gaohui Mao, Bingyan Guo, Shanshan Xie, Yurong Yang

**Affiliations:** College of Veterinary Medicine, Henan Agricultural University Zhengzhou 450000 China

**Keywords:** Fur seal (*Callorhinus ursinus*), *Toxoplasma gondii*, Isolation, ToxoDB#5, Avirulence

## Abstract

*Toxoplasma gondii* infects almost all warm-blooded animals, including marine mammals. Toxoplasmosis has been reported in wild and captive marine mammals in North America; however, no viable *T. gondii* strains have been isolated from northern fur seals. In this study, reproduction and *T. gondii* infection status were investigated in 10 northern fur seals (*Callorhinus ursinus*), from tissues collected from 2012 to 2024 in China. *Toxoplasma gondii* infections were determined by the modified agglutination test (MAT), PCR, immunohistochemical (IHC) staining, and isolation of the parasite by bioassay in mice. MAT was performed using placenta or tissue exudates to detect anti-*T. gondii* IgG antibodies. Four of the 10 seals had anti-*T. gondii* antibodies; *Toxoplasma gondii* DNA was detected by PCR in placenta tissues of two of these four animals, and *T. gondii* antibody positive reactions were observed in four seals by IHC. A viable *T. gondii* strain, TgFurSealCHn1, was isolated from placenta of one seal by bioassay in mice. In all, five seals had signs of *T. gondii* infection, and three of them had fetal stillbirth. One stillborn fetus had *T. gondii* nucleic acid detected by PCR, indicating potential vertical transmission of the parasite. Multilocus genetic typing of the TgFurSealCHn1 isolate revealed ToxoDB #5 genotype, which had demonstrated avirulence in Swiss Webster outbred mice, and the ROP18/ROP5 type was 2/2. ToxoDB #5 is the dominant genotype of wild terrestrial and marine mammals in North America. This is the first report of a viable *T. gondii* strain isolated from northern fur seal placenta.

## Abstract (fr)

*Toxoplasma gondii* infecte presque tous les animaux à sang chaud, y compris les mammifères marins. La toxoplasmose a été signalée chez des mammifères marins sauvages et captifs en Amérique du Nord; cependant, aucune souche viable de *T. gondii* n’a été isolée chez les otaries à fourrure du Nord. Dans cette étude, la reproduction et le statut infectieux de *T. gondii* ont été étudiés chez 10 otaries à fourrure du Nord (*Callorhinus ursinus*), à partir de tissus prélevés entre 2012 et 2024 en Chine. Les infections à *Toxoplasma gondii* ont été déterminées par le test d’agglutination modifié (TAM), la PCR, la coloration immunohistochimique (CIH) et l’isolement du parasite par bio-essai chez la souris. Le TAM a été réalisé à partir de placenta ou d’exsudats tissulaires afin de détecter des anticorps IgG anti-*T. gondii*. Quatre des 10 otaries présentaient des anti-*T. gondii;* l’ADN de *T. gondii* a été détecté par PCR dans les tissus placentaires de deux de ces quatre animaux, et des réactions positives aux anticorps anti-*T. gondii* ont été observées chez quatre otaries par CIH. Une souche viable de *T. gondii*, TgFurSealCHn1, a été isolée du placenta d’une otarie par bio-essai chez la souris. Au total, 5 otaries présentaient des signes d’infection à *T. gondii*, et 3 d’entre elles étaient mortes-nées. Un fœtus mort-né avait de l’acide nucléique de *T. gondii* détecté par PCR, indiquant une transmission verticale potentielle du parasite. Le typage génétique multilocus de l’isolat TgFurSealCHn1 a révélé le génotype ToxoDB #5, qui avait démontré une avirulence chez des souris non consanguines Swiss Webster, et le type ROP18/ROP5 était 2/2. ToxoDB #5 est le génotype dominant des mammifères terrestres et marins sauvages en Amérique du Nord. Il s’agit du premier rapport d’une souche viable de *T. gondii* isolée du placenta d’otarie à fourrure du Nord.

## Introduction

*Toxoplasma gondii* is an intracellular protozoan parasite that infects nearly all warm-blooded animals, including marine mammals [7, 10, 22]. Several studies have demonstrated that *T. gondii* is present in marine mammal species, including pinnipeds, cetaceans, sea otters, and polar bears [4, 7, 15, 22, 23, 35]. Infection typically occurs through ingestion of *T. gondii* sporulated oocysts from felines, tissue cysts from the definite host or intermediate host, or vertical transmission through the placenta [7]. Oocysts are the primary source of infection for *T. gondii*, either through direct ingestion or the ingestion of mechanical carriers such as bivalve mollusks, sardines, and anchovies, all of which can carry and concentrate oocysts [1, 18, 19, 21]. Marine mammals are considered sentinels of *T. gondii* oocyst pollution in marine environments [3, 5, 15, 20, 35]. Furthermore, vertical transplacental transmission of *T. gondii* has been described in chronically infected sea otters and Australian fur seals [14, 29]. However, the *T. gondii* infection status in fur seals is unknown, and the contribution of *T. gondii* infection to fetal loss is unknown. Therefore, the aim of this study was to explore whether *T. gondii* was present in the placenta, aborted fetuses, and other available tissues from fur seals.

## Materials and methods

## Ethics statement

All experiments were approved by the Institutional Animal Use Protocol Committee of Henan Agricultural University, China. All animals were handled in accordance with the Animal Ethics Procedures and Guidelines of China.

### Sample collection

A total of 10 northern fur seals (*Callorhinus ursinus*) from zoos in China were observed from 2012 to 2024. Fur seal case #2 was imported from the United States (Alaska) in 2010, and cases #1 and #3–10 were from domestic breeding in China. All of them were fed frozen, thawed, commercially sourced fish imported from other countries. Samples were collected from these fur seals, including ten fresh placentas, two fresh stillbirths, two fresh pups, one fresh male, and four female fur seals with multiple organs fixed in formalin (Table 1). The samples were sent to the Veterinary Pathology Laboratory of Henan Agricultural University for pathology diagnosis, etiological diagnosis, and the detection of *T. gondii*. In addition, placental exudates were centrifuged and collected.

**Table 1:** Background and *T. gondii* infection in fur seals (*Callorhinus ursinus*) from China (2012–2024).

Case #	Sex, age (years)	Date	Fertility	Samples	Clinical features	Pathological number and findings	MAT^a^	PCR^b^	IHC ^c^	Mice Bioassay ^d^
1	Male, 4	6/22/2016	NA	Multiple organs	Died. Fought for mates, lived in cage and did not dive into water for six days.	#2220: Respiratory distress, myocardial necrosis; hepatic steatosis, gastroenteritis catarrhalis (metabolic acidosis)	<1:25	–	–	NA
2	Female, adult	6/2012	Viable fetus named case #3	NA						
		6/2013	Unproduced	NA						
		6/2014	Viable fetus	NA						
		6/2015	Stillborn fetus	NA						
		6/5/2016	Viable fetus	Placenta	Healthy.	#2218: Hematoidin crystals	1:128	–	–	0/4
		6/19/2017	Stillborn fetus	Placenta and fetal tissue	Fetus was 4.25 kg, 54 cm length, fin necrosis	#2511: Blood vessels in the placenta were calcified	Placenta 1:64; Fetal: < 1:25	Placenta – Fetal:–	Placenta – Fetal:–	0/3
		5/28/2018	Viable fetus	Placenta		#2511-4: Hematoidin crystals	1:256	–	–	NA
		2019	Viable fetus	NA						
		2020	Viable fetus	NA						
		2021	Viable fetus	NA						
		6/17/2022	Viable fetus	Placenta		#3445: Hematoidin crystals	1:2048	Placenta +	Placenta +	1/8
		6/5/2023	Viable fetus	Placenta		#3559: No obvious abnormality	<1:25	–	Placenta +	0/4; 0/1 IFN**-γ**^**−/−**^
		6/2024	Viable fetus	NA						
3 offspring from case #2	Female, Adult	6/17/2016	Viable fetus	Placenta	Firstborn	NA	<1:25	–	NA	NA
		6/27/2019	Viable fetus	Placenta		#2955: No obvious abnormality	1:256	–	–	0/3
		6/2024	Viable fetus	NA						
4	Female, Adult	6/29/2017	Viable fetus	Placenta		NA	<1:25	–	NA	0/2
		6/29/2023	Viable fetus	Placenta		#3568: Hematoidin crystals	<1:25	–	Placenta +	0/5
		9/2/2023	Deceased pup	Multiple organs of the pup	Female, 2 months, 6.78 kg, diarrhea	#3600: Suppurative bronchopneumonia; hepatic and renal insufficiency, hemorrhagic necrotizing splenitis; necrotizing enteritis	<1:25	–	–	NA
		6/2024	Viable fetus	NA						
5	Female, Adult	2022	Stillborn fetus	NA						
		2023	NA	NA	Healthy.					
		7/1/2024	Miscarriage in February 2024	Multiple organs ^e^	Died. Cough. The weight was 45.36 kg, and the length was 160 cm	#3790: Multiple organ microcirculation disorder, thrombosis, hypoxia; pulmonary sarcoma; uterine leiomyosarcoma; ovarian atrophy; cardiomyocyte necrosis	NA	NA	–	NA
6	Female, 6	7/18/2024	NA	Multiple organs ^e^	Died. The liver was partially yellow; pulmonary carnification; atrophic kidney with grey-white nodules.	#3802: Multiple arteriosclerosis in multiple organs, histocytoma of kidney, uterine fibroids. The mucosal epithelium of the pulmonary airway system was seriously damaged (vitamin A deficiency), necrotizing enteritis and necrotizing gastritis	NA	NA	–	NA
7	Female, Adult	8/4/2024	Miscarriage in February 2024	Multiple organs ^e^	Died. Stillborn fetus was positive for *T. gondii* by serology, 1:64.	#3814: Multiple organ infectious granuloma, *Mycobacterium tuberculosis*	NA	NA	Lungs +	NA
8	Female	9/12/2024	Stillborn fetus	Placenta, fetal tissues	Died.	#3841: Placental focal necrosis, fetal cardiac insufficiency, acute liver injury	Placenta 1:64 Fetal:<1:25	Placenta + Fetal:+ ^f^	Placenta + Fetal –	0/5
9	Female	9/28/2024	Pup	Multiple organs ^e^	Died. 56 days old, diarrhea, dyspnea	#3859: Acute suppurative pneumonia; acute enteritis; hypoproteinemia; multiple organs hypoplasia	NA	NA	–	NA
Percent	50% (5/10)				15	50% (7/14)	21% (3/14)	29% (5/17)	13% (1/8)	Percent Positive cases
Positive cases	Case #2, #3, #4, #7 and #8					Cases #2, #3, #7, and #8	Cases #2 and #8	Cases #2, #4, #7, and #8	Case #2	

*Note*: Fur seal case #2 were imported from the United States (Alaska) in 2010, and cases #1, and 3–10 were from domestic breeding in China.

NA: not available for tissues; means the experiment was not performed.

“–”: no anti-*T. gondii* antibodies or *T. gondii* parasites.

a : MAT: modified agglutination test; ^b^ PCR: polymerase chain reaction; ^c^: IHC: immunohistochemistry; ^d^: mouse bioassay: Swiss mice; ^e^: formalin fixation. ^f^: *T. gondii* nucleic acid was detected in the placenta, fetal heart, liver, and lungs.

### Detection of *T. gondii* antibodies in fur seals

The placental and heart exudates from fur seals were tested for *T. gondii* antibodies using a modified agglutination assay (MAT) [8]. First, we tested whether the sample was infected with *T. gondii* by two titers (1:25 and 1:200) on the sample received date. Then, retrospective study was performed on *T. gondii* positive samples in 2024 (double dilution from 1:16 to endpoint dilution). The MAT antigen was obtained from the University of Tennessee Research Foundation (Knoxville, TN, USA). Positive (VEG-infected mice) and negative (*T. gondii*-free mice) control serum samples were used in each 96-well U plate. The MAT cut-off titer was 1:25 [7, 20].

### Pathology analysis

Fur seal tissue samples were fixed in formalin. Paraffin sections were prepared using conventional techniques, hematoxylin and eosin (H&E) staining, and immunohistochemistry (IHC) [36]. The primary antibody used was a rabbit anti-*T. gondii* polyclonal antibody. Anti-rabbit IgG conjugated with Horseradish Peroxidase (HRP)/3,3'-Diaminobenzidine (DAB) (IHC detection kit, ab64261, Abcam, Cambridge, United Kingdom) was used as the secondary antibody. Placental tissue sections of sheep infected with *T. gondii* VEG strain (provided by Dr. Dubey) were used as positive controls for IHC staining. *Toxoplasma gondii-*free mice brains were used as negative controls. The primary antibody was diluted to 1:3,000 and incubated overnight at 4 °C. The secondary antibody was treated at 37 °C for 15 min. The signal was amplified with streptavidin-peroxidase and then visualized with DAB under a microscope. Finally, it was counterstained with hematoxylin, dehydrated, and mounted in neutral balsam.

### DNA extraction from fur seal tissues and detection of *T. gondii* nucleic acid by PCR

Genomic DNA was extracted from the placenta, heart, liver, spleen, lungs, kidneys, and other tissue samples of the fur seals using a commercial kit (Tiangen Biotech, DP304, Beijing, China). *Toxoplasma gondii-*specific primers TOX5 and TOX8 were used to detect *T. gondii* nucleic acids [28, 37]. The PCR reaction system was 25 μL, which contained 12.5 μL of 2× PCR Mix (GDSBio, Guangzhou, China), 2 μL of primers (with a primer concentration of 0.2 μM), 2 μL of target DNA, and 8.5 μL of ddH_2_O. The PCR reaction involved initial denaturation at 94 °C for 5 min, denaturation at 94 °C for 1 min, annealing at 60 °C for 1 min, extension at 72 °C for 1 min, for a total of 35 cycles, and finally extension at 72 °C for 10 min. The PCR product was approximately 450 bp, which indicated the presence of *T. gondii* pathogen nucleic acid in tissues. During DNA extraction and PCR amplification, a negative control (commercial negative samples from a *T. gondii* nucleic acid detection kit, Product *ID*: 25T, Shanghai Yan Qi Biological Technology Company, Shanghai, China) and a positive control (brain tissues from Me49 strain-infected mice) were included for each experimental batch.

### Isolation of viable *T. gondii* from tissues of fur seals using mouse bioassay

Two methods were used to process fur seal tissue for mouse bioassay. One was taking approximately 50 g of fur seal tissue and digesting it with hog pepsin, and the other was taking approximately 5 g of fur seal tissue and grinding it thoroughly [7]. Tissue fluid was inoculated subcutaneously into Swiss mice (*n* = 2−5) and/or interferon gamma knockout mice (*IFN-γ*^*−/−*^, *n* = 1). The mice were observed and recorded (appetite, activity level) every day. *Toxoplasma gondii* parasites were detected in the brain and lungs of the dead mice. The blood of the surviving mice was collected at 30 days and 60 days post-inoculation (DPI), and their *T. gondii* infection status was determined *via* MAT (titer: 1:25, 1:200). If *T. gondii* tachyzoites, cysts, or antibodies were observed, the brain, heart, spleen, and other tissues were ground and inoculated into a new group of mice to preserve and isolate the viable strain.

### Cell culture, genotyping, and morphological observation of *T. gondii* from fur seals

The tissue homogenates (brain, heart, lungs, spleen, and membranous lymph nodes) of *T. gondii-*positive mice were inoculated into Vero cells, and the fluid was changed twice per week. DNA was extracted and collected from *T. gondii* tachyzoites using commercial DNA kits, and the genotypes of *T. gondii* were determined using restriction fragment length polymorphism-polymerase chain reaction (RFLP-PCR) with 10 genetic markers [32]. *Toxoplasma gondii* virulence factors were identified by genotyping ROP5 and ROP18 polymorphisms [31]. *Toxoplasma gondii* reference DNA *(n* = 8) was included in the batches.

When numerous parasitophorous vacuoles were observed, a glutaral-paraformaldehyde mixture was added to the cell culture flask for pre-fixation, after which the cells were scraped off with a cell scraper and centrifuged, with the supernatant being discarded. The glutaral-paraformaldehyde mixture was then added to the precipitate for fixation, and the samples were sent to the Henan University of Chinese Medicine for transmission electron microscope (TEM) section production.

### Evaluation of the virulence of *T. gondii* isolated from fur seals using Swiss mice

*Toxoplasma gondii* tachyzoites were collected from the cell culture, counted, and diluted 10-fold to 10^4^, 10^3^, 10^2^, 10^1^, 10^0^, and <1 per mL. Following this, the diluted tachyzoites were inoculated intraperitoneally into five Swiss mice in each group, and the mice were checked daily [26]. At 30 and 48 DPI, the blood of mice was collected and tested for *T. gondii* IgG antibodies using MAT with titers between 1:25 and 1:200. This study could not be conducted over a longer period because of the COVID-19 epidemic in China, during which the laboratory was closed (48 DPI: 9 November 2022). The mice were euthanized at 48 DPI, and *T. gondii* cysts in the brain were counted [9]. In brief, the whole mouse brain was homogenized with 1 mL of saline (0.85% NaCl) and tissue cysts were counted microscopically in 50 μL homogenate, and the count was multiplied by 20 to obtain the number of tissue cysts per brain. Two replicates were performed for each mouse brain. Tissue samples from mice infected with *T. gondii* were fixed in formalin. Virulence was assessed based on the percentage of dead mice among the *T. gondii*-infected mice [26].

### Statistical analysis

Data were expressed as the mean ± SEM using the GraphPad Prism 8.0, followed by one-way analysis of variance to test whether there were significant differences, with *p* < 0.05 considered statistically significant.

## Results

### The cause of death and *T. gondii* infection in fur seals

**Figure 1:**
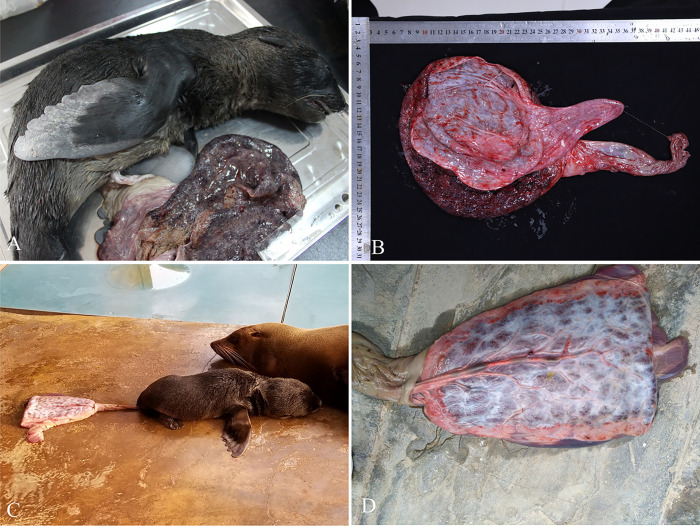
Morphology of the placenta and samples from fur seal #2. A: Stillborn and placental tissue in 2017. B: Placenta in 2022. C: Zonary and belt-shaped placenta, dam, and its pup. D: Magnified C: surface of the chorioallantoic membrane.

**Figure S1:**
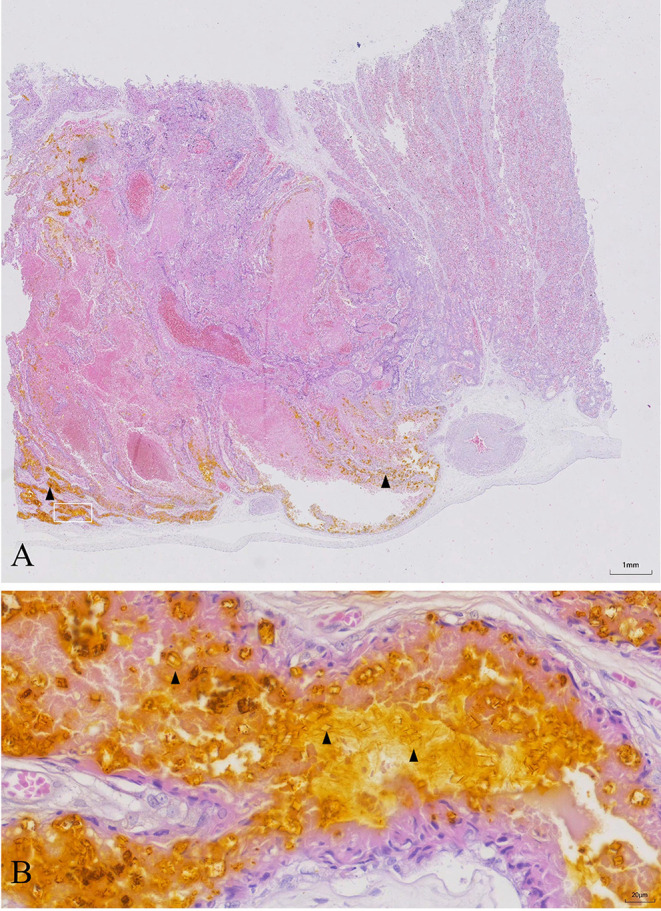
Morphology of fur seal placenta and hematoidin crystal distribution as shown by microscopy. A: Large quantity of hematoidin crystals (trigon) were observed in the chorioallantoic membrane (fetal facial placenta). B: Magnified image of A (white square); hematoidin crystals are yellow rhombic plates (trigons).

The background information, clinical symptoms, pathological changes, and *T. gondii* infection status of the fur seals (*n* = 10) are summarized in Table 1. The fur seal placenta was found zonary and belt-shaped (Fig. 1). Large quantities of hematoidin crystals (yellow rhombic plates, bilirubin) were observed in the intervillous space of the chorioallantoic membrane (Figure S1).

Three fur seals (cases #2, #3, and #4) had no clinical abnormality and were alive. In the seven fur seals (cases #1, #5, #6, #7, #8, #9, and #10) that underwent detailed pathological autopsy and histopathological examination, *T. gondii* infection was not considered the primary cause of death, but it may have contributed to the death of one fur seal (case #7). Among them, one male (case #1) had died of hydropenia (kept out of water cages for 6 days), two pups (case #9 and the pup of case #4, both 2 months old) died of diarrhea, and four adult females died of the following: *Mycobacterium tuberculosis* infection (case #7), vitamin A deficiency (case #6), pleuropneumonia (case #10), and multiple tumors (case #5). Based on detailed *T. gondii* parasite examinations using PCR, MAT, and IHC data, five cases (cases #2, #3, #4, #7, and #8) were exposed to this parasite from the ten cases. However, three cases (cases #2, #7, and #8) resulted in stillbirths, while the other cases (cases #3 and #4) had viable fetuses.

Fur seal case #1, which was male, was placed into a cage for six days after it fought with the other fur seals, where it died of hydropenia. Microscopically, necrotizing myocarditis, hepatic steatosis, and gastroenteritis catarrhalis were observed.

Fur seal case #2 had a history of abnormal reproduction every other year from 2012 to 2015, and a follow-up study was performed. A total of five placentas (2016, 2017, 2018, 2022, and 2023) from case #2 were collected. In 2017, fur seal case #2 gave birth to a stillborn fetus in the third trimester of pregnancy. However, there was clear grey necrosis at the end of the fin of the stillborn fetus (Fig. 1). Microscopically, many blood vessel calcifications were found to have occurred in the placenta, and it was speculated that the blood vessels were blocked, resulting in anemia at the end of the fetal limb.

The mother of fur seal case #3 (born 2012) was fur seal case #2. Fur seal case #3 had its firstborn in 2016, and then birthed a second pup in 2019; it had no history of abortion.

Fur seal case #4 gave birth to a baby that died at 66 days of age in 2023. Pathology examination revealed suppurative bronchial pneumonia, hepatic and renal insufficiency, hemorrhagic necrotizing splenitis, and necrotizing enteritis.

Fur seal case #5 showed pulmonary sarcoma, liver insufficiency, thrombosis in the liver, spleen, kidney, and intestines, as well as uterine leiomyosarcoma, necrotizing adrenal inflammation, necrotizing thyroiditis, and necrotizing myocarditis. It had an abortion in 2022.

Arteriosclerosis in multiple organs (the uterus, spleen, kidney, stomach, and intestine) was observed in fur seal case #6 based on pathology. Furthermore, histiocytoma in the kidney, uterine fibroids, and lesions in the mucosal epithelium of the pulmonary airway system (vitamin A deficiency) were observed in case #6.

Fur seal case #7 showed multi-organ infectious granuloma (heart, lung, liver, intestine, spleen, uterus, stomach, and lymph nodes) and tested positive for acid-fast staining, which indicated *Mycobacterium tuberculosis* infection, confirmed by PCR and sequencing. It had a history of abortion, and the stillborn fetus was seropositive for *T. gondii*.

Fur seal case #8 showed placental focal necrosis, fetal cardiac insufficiency, and acute liver injury. Additionally, *T. gondii* was detected by MAT, PCR, IHC in the placental tissue of case #8, and *T. gondii* nucleic acid was detected in the heart, liver, and lungs of its stillborn fetus by PCR.

The pup of fur seal case #9 showed acute suppurative pneumonia, hypoproteinemia, acute enteritis, and multiple organ hypoplasia.

Fur seal case #10 exhibited fibrinous pleuropneumonia.

### Detection of antibodies and pathogen *T. gondii* in fur seals

In this study, ten zoo-housed fur seals were tracked and monitored for their reproduction and *T. gondii* infection status. Among them, six of nine (67%, 95%CI: 35.09%–88.27%) females (case #2, #4, #5, #7, #8, and #9) had a history of poor reproduction. Based on their serology or etiology, five fur seals (case #2, #3, #4, #7, and #8; 50%, 95%CI: 23.66%–76.34%, 5/10) showed evidence of exposure to *T. gondii*.

Ten placental exudates, two heart exudates, and three fetal exudates from six adult fur seals (cases #1, #2, #3, #4, #7, and #8) were tested, and seven samples (50%, 95%CI: 26.80%–73.20%, 7/14) showed the presence of *T. gondii* antibodies using MAT; these samples belonged to four fur seals (cases #2, #3, #7, and #8). The exudates from four placentas of fur seal case #2 tested positive for anti-*T. gondii* antibodies, with titers of 1:128 (2015), 1:64 (2017), 1:256 (2018), and 1:2048 (2022). However, this fur seal produced three healthy fetuses and only one stillborn fetus in 2017. In 2023, the anti-*T. gondii* antibody titer in the placental exudate of fur seal case #2 was below 1:25. In 2012, fur seal case #2 gave birth to a healthy female fur seal, named fur seal case #3. The placental exudate of case #3 tested positive for *T. gondii* antibodies with titers of 1:256 (2019) and 1:64 (2025). However, it delivered four healthy fetuses in 2016, 2019, 2024, and 2025.

**Figure 2:**
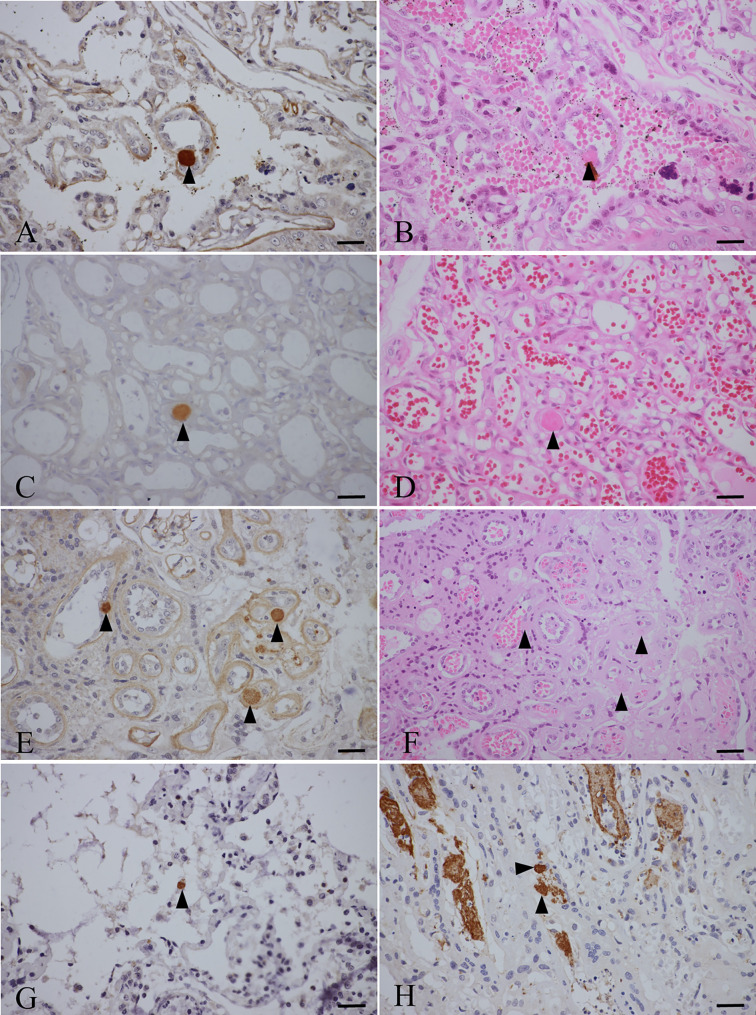
Morphology of *T. gondii* in the tissues of the fur seals. A–D: *T. gondii* antibody positive reaction (trigon) in the placentas of fur seal #2. A and C show IHC staining, while B (P#3445) and D (P#3559) show HE staining of serial sections A and C, respectively. E, F: *T. gondii* antibody positive reaction (trigon) in placentas of fur seal #4. E shows IHC staining, and F (P#3568) shows HE staining of the E serial section. G: *T. gondii* antibody positive reaction in the lung of fur seal #7 (P#3814) shown by IHC staining (trigon). H: *T. gondii* antibody positive reaction in the placenta of fur seal #8 (P#3841) shown by IHC staining (trigon). Bar = 50 μm.

Eight placentas, multiple organs from seven fur seals, and two fetal tissues were examined for *T. gondii* antigens using IHC, and *T. gondii* antibody positive reactions were observed in five samples (four placentas and a lung) (29%, 95%CI: 12.99%–53.43%, 5/17); these samples belonged to four fur seals (cases #2, #4, #7, and #8) (Fig. 2). *Toxoplasma gondii-*like bradyzoites were mainly distributed in the vascular endothelial cells of placental trophoblasts.

Among the ten placentas, multiple organs from two fur seals, and two fetal tissue samples, 21% *T. gondii* nucleic acid (95%CI: 6.84%–48.32%, 3/14) was detected by PCR in the placental tissue of fur seal case #2 in 2022, as well as in the placenta and stillborn fetal heart, liver, and lung of case #8 (Table 1).

### Isolation, genotyping, and transmission electron microscopy of *T. gondii*

**Figure 3:**
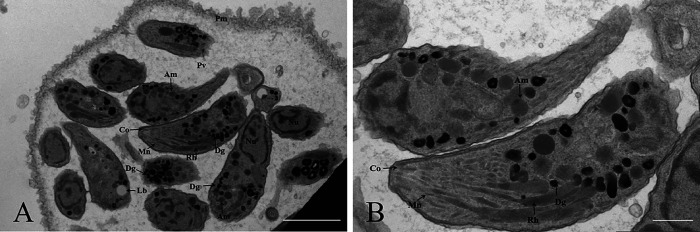
Morphology of tachyzoites of TgFurSealCHn1 in cell culture under transmission electron microscopy. A: A group of TgFurSealCHn1 tachyzoites within the parasitophorous vacuolar membrane. B: Magnified image of A. Am: amylopectin granule, Co: conoid, Dg: electron-dense granule, Lb: lipid, Mn: microneme, Nu: nuclei of daughter tachyzoites, Pm: parasitophorous vacuolar membrane, Pv: parasitophorous vacuole, Rh: rhoptry. Bar = 2 μm.

Eight placental tissue homogenates (2016–2024) from four fur seals (cases #2, #3, #4, and #8) were inoculated into Swiss mice or *IFN-γ*^–/–^ mice (Table 1). *Toxoplasma gondii* antibodies (MAT titer was ≥1:200) were detected in Swiss mouse M#43 in the Tox#12-9 group (inoculated placenta obtained in 2022 from case #2) at 30 DPI, and many cysts (*n* = 1,180) were found in the brain on 54 DPI. The mouse brain *T. gondii* cysts were successfully propagated in cell culture (19 DPI) and were named TgFurSealCHn1. Abundant electron-dense granules (3.74 ± 0.55, *n* = 62) and amylopectin granules (2.63 ± 0.33, *n* = 62) were observed in *T. gondii* tachyzoites through transmission electron microscopy (Fig. 3).

**Figure 4:**
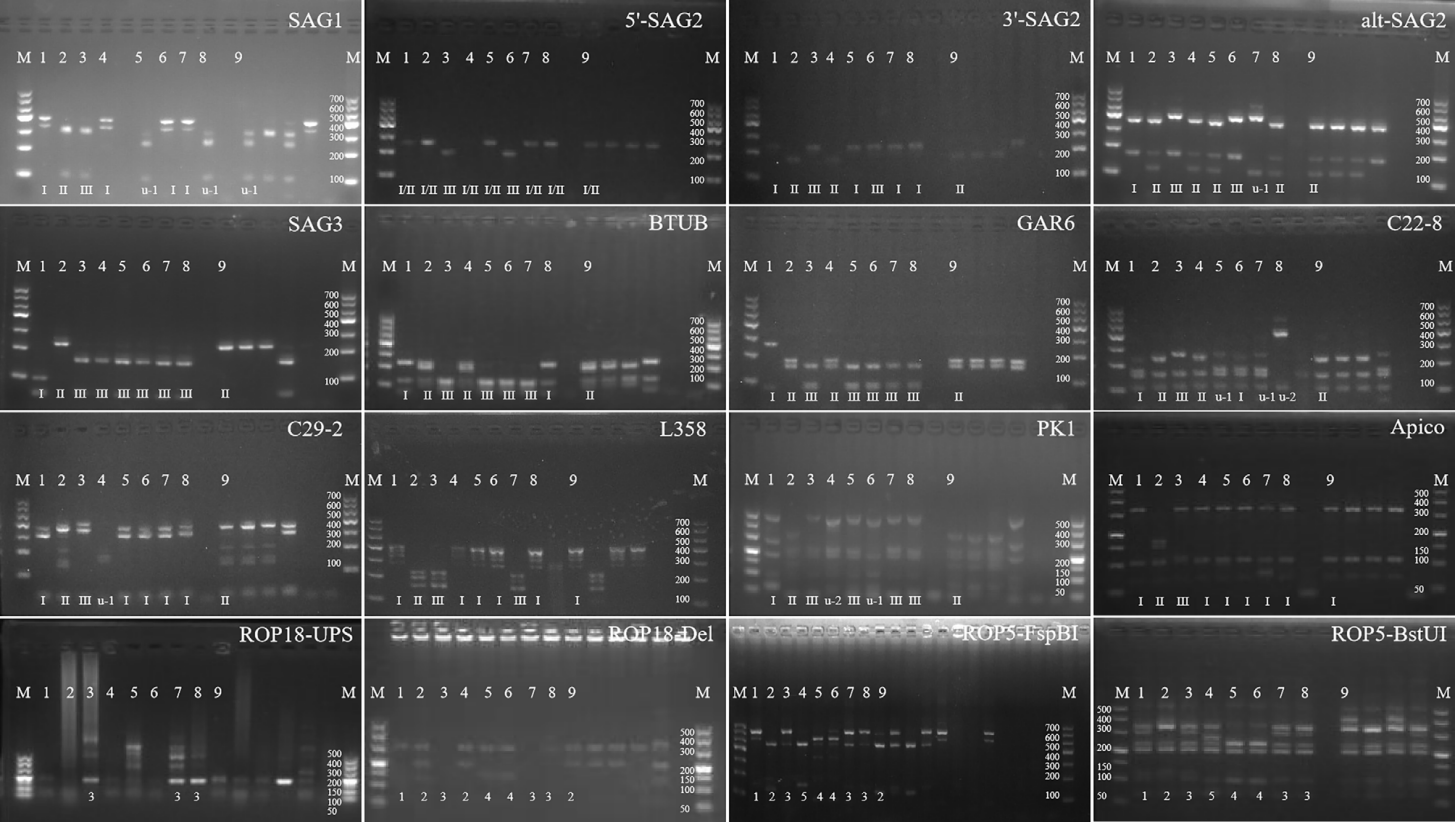
Genotyping of *T. gondii* TgFurSealCHn1 strain isolated from fur seals. 1: GT1, 2: PTG, 3: CTG, 4: TgCgCal, 5: MAS, 6: TgCatBr5, 7: TgCatBr64, 8: TgToucan (TgRsCr1), 9: TgFurSealCHn1, and M: Marker.

The genotype of TgFurSealCHn1 was ToxoDB #5, which was determined using PCR-RFLP with 10 markers. In addition, the allele types of ROP18 and ROP5 genes were 2/2 (Fig. 4, Table 2).

**Table 2:** Genotypes of *T. gondii* isolates from fur seal according to PCR-RFLP of 10 markers and virulence proteins.

Isolated ID	SAG1	(3′ + 5′) SAG2	Alt SAG2	SAG3	BTUB	GRA6	C22-8	C29-2	L358	PK1	Apico	ROP18	ROP5	ToxoDB Genotype
GT1, reference	I	I	I	I	I	I	I	I	I	I	I	1	1	#10
PTG, reference	II/III	II	II	II	II	II	II	II	II	II	II	2	2	#1
CTG, reference	II/III	III	III	III	III	III	III	III	III	III	III	3	3	#2
TgCgCa1, reference	I	II	II	III	II	II	II	u-1	I	u-2	I	2	5	#66
MAS, reference	u-1	I	II	III	III	III	u-1	I	I	III	I	4	4	#17
TgCatBr5, reference	I	III	III	III	III	III	I	I	I	u-1	I	4	4	#19
TgCatBr64, reference	I	I	u-1	III	III	III	u-1	I	III	III	I	3	3	#111
TgRsCr1, reference	u-1	I	II	III	I	III	u-2	I	I	III	I	3	3	#52
TgFurSealCHn1	u-1	II	II	II	II	II	II	II	I	II	I	2	2	#5

Other fur seal placentas (cases #3, #4, and #8) failed to isolate viable *T. gondii*.

### Evaluation of the virulence of the TgFurSealCHn1 strain in Swiss mice

After inoculation with tachyzoites from different gradients of TgFurSealCHn1, the survival times of Swiss mice infected with *T. gondii* were recorded (Table 3). Ten tachyzoites infected 100% of the mice (MAT titer, ≥1:200). Under 48 days observation period, the survival rates of mice were 20%, 80%, 100%, and 100% for the 10^4^, 10^3^, 10^2^, and 10 tachyzoites, respectively. Cumulative mouse mortality was 7% after infection of 10 – 10^3^ tachyzoites. The number of cysts in the mouse brains was examined, and there was no significant difference among the different tachyzoite gradient groups at 48 DPI (*p* > 0.05).

**Table 3:** Evaluation of the virulence of *T. gondii* TgFurSealCHn1 strain in Swiss mice (M ± SE).

No. of tachyzoites	No. infected/No. inoculated (%)	Days of survival/number of mice	No. of brain cysts*
10^4^	5/5(100%)	21 DPI/1, 26 DPI/1, 31 DPI/1, 44 DPI/1, **≥**48 DPI/1	174 ± 128
10^3^	5/5(100%)	15 DPI/1, **≥**48 DPI/4	42 ± 27
10^2^	5/5(100%)	**≥**48 DPI/5	12 ± 6
10^1^	5/5(100%)	**≥**48 DPI/5	50 ± 31
10^0^	2/5(40%)	**≥**48 DPI/5	0
<1	0/5(0)	**≥**48 DPI/5	0
Negative control	0/5(0)	**≥**48 DPI/5	0

DPI: days post-infection.

The number of mice injected per group is 5.

The dosages 1 × 10^3^, 1 × 10^2^, and 1 × 10^1^ are included for calculation.

The cumulative mortality is 1/(4 + 5+5) = 7%.

*There was no significant difference among the different tachyzoite gradient groups (*p* > 0.05).

## Discussion

In the present study, *T. gondii* was found to be highly infectious in captive fur seals (50%), consistent with reported infection patterns in other marine animals [7, 20]. Reported exposure rates are 25.9% for sea otters (IFA cut-off 1:320) [2], 86.3% for dolphins (MAT cut-off 1:25) [27], and 32.8% for sea lions (IFA cut-off 1:40) [3]. Notably, *T. gondii* was detected in 7.5% of third trimester abortions in Australian fur seals [14]. Here, the infection rate of *T. gondii* in fur seals was higher than that of food animals of local origin (*p* < 0.05) [6].

Direct evidence of *T. gondii* infection in fur seal was observed by isolation of viable strain and was named TgFurSealCHn1. A viable strain of *T. gondii* was successfully isolated from fur seal case #2 placenta (2022) using a mouse biological method. Case #2 already had anti-*T. gondii* antibody in 2016 (1:128), and the placental exudate showed a *T. gondii* titer of 1:2,048 in 2022; *T. gondii-*like parasites and nucleic acids were also detected in placental tissue in 2022 by IHC and PCR. However, the puppy showed no abnormalities and survival. These results indicate that endogenous *T. gondii* vertical transmission during pregnancy or exogenous oocysts reinfection occurred in 2022. The former phenomenon has been reported in sheep [7, 16]; however, there are only a few reports on marine mammals. Furthermore, the serological reaction from the placental exudate of case #2 was inconsistent from 2016 to 2023. MAT can be used to detect IgG antibodies, with a continued increase in antibody levels, indicating the activation and proliferation of *T. gondii,* and decreased antibody levels indicating that the parasites may have been eliminated [8]. This phenomenon of fluctuating *T. gondii* antibody levels was also observed by Martins *et al.* in fur seals by MAT [20]. Interestingly, case #2 was serologically negative in placental exudate in 2023, yet HE and IHC staining still detected *T. gondii-*like parasites in the placenta. Cattle have shown transient antibody responses to *T. gondii* infection because they developed an effective immune response that may facilitate *T. gondii* elimination [11, 12]. Unlike other animals, which exhibit a sustained antibody response, the fur seal could have eliminated *T. gondii* and shown serologically negative results. Whether fur seals have a unique immune response to *T. gondii* infection (like that in cattle) needs to be further explored. Fur seals case #2 and case #4 had *T. gondii-*like parasites in the placenta in 2023; however, the serological reaction was negative. This phenomenon was associated with lower antibody titers in placental tissue exudate than in serum, or it could indicate that case #2 and case #4 were at the stage of acute *T. gondii* infection. Serum samples from these animals should be collected for further *T. gondii* confirmation, if possible. The *T. gondii* antibody in the placental exudate of fur seal case #3 was lower than 1:25 in 2016, but increased to 1:256 in 2019. This means that case #3 was postnatally exposed to *T. gondii* and indicates that cat *T. gondii* oocysts may contaminate the food or environment of the zoo. Oocysts can survive even at −20 °C for 28 days, indicating that standard freezing conditions are insufficient to eliminate *T. gondii* infectivity in frozen fish products [13].

Genotyping of TgFurSealCHn1 suggested potential *T. gondii* transmission *via* imported frozen fish or fur seal. The TgFurSealCHn1 was identified as ToxoDB PCR-RFLP genotype #5. ToxoDB #5 (along with ToxoDB #4 and #5, collectively forming haplotype 12) was the dominant type in wildlife from North America, including marine mammals (*e.g.*, sea otters), but rarely in animals from the rest of the world and domestic animals [7, 10]. Studies have shown that different genotypes of *T. gondii* have important relationships with geographic regions [7]. The epidemic genotype of *T. gondii* is ToxoDB #9 in China, and only one strain of the ToxoDB#5 genotype has been reported in a caracal from China, which may have been spread from North America by marine animals, feral birds, or sea trade routes [24]. The transmission process of the *T. gondii* ToxoDB #5 strain in China is unknown. Fur seals in zoos from China were not checked for *T. gondii* infection status when imported from Alaska. Frozen sea fish are the main food for these animals. According to the distribution of ToxoDB #5, case #2, from which TgFurSealCHn1 was isolated, was a plausible carrier of the pathogen upon import or infection from oocysts in frozen fish imported from North American.

*T. gondii* ROP18 and ROP5 allele types were associated with virulence in mice. The allele types of virulence genes ROP18 and ROP5 (2/2) was predicted to be non-lethal in mice [31], which is consistent with the virulence evaluation of TgFurSealCHn1 in Swiss mice (survival rate 77%, 17/22, 48DPI) (Table 3). The mouse has been used as the main animal model for determining the virulence of *T. gondii* strains. Epidemiologic data suggest a potential association between *T. gondii* virulence in mice and disease manifestations in humans, and other susceptible animals [7, 26]. However, the severity of the toxoplasmosis following natural infection varies in intermediate hosts, and it is particularly challenging in captive or wild animals, in which detailed etiology and pathology surveys for *T. gondii* infection are rare [7, 35]. ToxoDB #5 *T. gondii* isolates (*n* = 117) from sea otters have been verified to be avirulent through mice assays, although most sea otters died of toxoplasmosis in these studies [30, 33, 34].

Vertical transmission of *T. gondii* in fur seals was proved in cases #2, #8, and #7 by serology, IHC, PCR, and the isolation of viable parasites from placenta. Vertical transmission of *T. gondii* causes fetal loss in several marine species, including dolphins and seals [17, 25]. *Toxoplasma gondii* transplacental transmission in sea otters was proved in a chronically infected dam by serology, IHC, and the isolation of viable parasites from the fetal brain [29, 30]. However, evidence of vertical transmission by isolating viable parasites from other marine mammals has not been reported. Here, *T. gondii* transplacental transmission in chronically infected fur seal case #2 was verified by serology (1:2018), IHC (placenta), PCR (placenta), and isolation of viable parasites from the placenta. Both the dam and fetus survived. In addition, the stillborn fetus and placenta from fur seal case #8 were proven to be infected with *T. gondii* by serology (dam 1:64, fetal <1:25), IHC (placenta positive, fetus negative), and PCR (placenta positive, fetal heart, liver, and lung positive), and the dam survived. Fur seal case #7 miscarried, and the aborted fetus was found to be positive for *T. gondii* by serology, and *T. gondii* tachyzoites were found in the lungs of fur seal case #7 by IHC, indicating possible vertical transmission of *T. gondii*.

Marine mammals are susceptible to *T. gondii* and are good sentinels for detecting marine pollution. Most *T. gondii* infections in people and animals are subclinical. However, sea otters are the most susceptible mammals in the United States [7, 10]. *Toxoplasma gondii* infections in sea otters can directly cause mortality due to meningoencephalitis [10, 34]. However, *T. gondii* infections were not lethal for fur seals (*n* = 5) in this study, although they were associated with abortion (3/5). Fur seals do not prey on the intermediate hosts of *T. gondii*, but they could be infected by the ingestion of oocysts from land sources or by ingesting mechanical transmitters from cold-blooded marine animals. In this survey, the high *T. gondii* infection rate in fur seals indicates that more attention should be paid to imported animals, frozen fish food, and oocyst-contaminated environments. The present study had several limitations. The first was the limited number of serum and tissue samples. The second was that the impact of *T. gondii* on marine mammals and the human care marine environment is not well understood. In the future, exploring the infection status of marine mammals and evaluating contamination by *T. gondii* oocysts will be of great significance for the health of marine animals and humans.

## Conclusions

In the present study, *T. gondii* infection in chronically infected fur seal case #2 was verified by serology (1:2018), IHC (placenta), PCR (placenta), and isolation of viable parasites from the placenta. Genotyping analysis of this isolate indicates a probable North American origin and suggests vertical transmission of *T. gondii* in fur seals. Future follow-up studies on fur seal #2 and its offspring, including fur seal #3 may provide stronger support for these conclusions.

## Supplementary material

The supplementary material for this article can be found at https://www.parasite-journal.org/10.1051/parasite/2025045/olm.
